# Abnormal left ventricular systolic reserve function detected by treadmill exercise stress echocardiography in asymptomatic type 2 diabetes

**DOI:** 10.3389/fcvm.2023.1253440

**Published:** 2023-10-19

**Authors:** Yuyou Duan, Luwei Ye, Qinglan Shu, Yu Huang, Hongmei Zhang, Qingfeng Zhang, Geqi Ding, Yan Deng, Chunmei Li, Lixue Yin, Yi Wang

**Affiliations:** ^1^ School of Clinical Medicine, Southwest Medical University, Luzhou, China; ^2^ Ultrasound in Cardiac Electrophysiology and Biomechanics Key Laboratory of Sichuan Province, Sichuan Provincial People's Hospital, University of Electronic Science and Technology of China, Chengdu, China

**Keywords:** asymptomatic type 2 diabetes mellitus, treadmill exercise stress echocardiography, two-dimensional speckle-tracking imaging, left ventricular reserve function, serum biological parameters

## Abstract

**Aims:**

Subclinical left ventricular (LV) dysfunction may occur in T2DM patients at the early asymptomatic stage, and LV reserve function is a sensitive index to detect subtle LV dysfunction. The purpose of our study is (1) to assess the LV reserve function using treadmill exercise stress echocardiography (ESE) in asymptomatic type 2 diabetes mellitus (T2DM) patients; (2) to explore the link of serum biological parameters and LV reserve function.

**Methods:**

This study included 84 patients with asymptomatic T2DM from September 2021 to July 2022 and 41 sex- and age-matched healthy controls during the corresponding period. All subjects completed treadmill ESE, LV systolic function-related parameters such as global longitudinal strain (GLS) and systolic strain rate (SRs), as well as diastolic function-related parameters such as E wave (E), early diastolic velocity (e′), E/e′ ratio, early diastolic SR (SRe), and late diastolic SR (SRa) were compared at rest and immediately after exercise. The difference between LV functional parameters after treadmill exercise and its corresponding resting value was used to compute LV reserve function. In addition, the associations of LV reserve function and serum biological parameters were analyzed.

**Results:**

Patients with T2DM did not significantly vary from the controls in terms of alterations in LV diastolic reserve measures, the changes of LVGLS and SRs (ΔGLS: 2.19 ± 2.72% vs. 4.13 ± 2.79%, *P *< 0.001 and ΔSRs:0.78 ± 0.33 s^−1^ vs. 1.02 ± 0.28 s^−1^, *P *< 0.001) in the T2DM group were both lower than those in the control group. Glycated hemoglobin (HbA1c), N-terminal pro-brain natriuretic peptide (NTproBNP), waist circumference, and high-sensitive C-reactive protein (hsCRP) were identified as independent predictors of LV systolic reserve by stepwise multiple linear regression analysis.

**Conclusion:**

LV systolic reserve function, as measured by pre- and post-exercise differences in GLS and SRs were significantly impaired in patients with asymptomatic T2DM, whereas diastolic reserve remained normal during exercise and was comparable to that of the control group. This was different from previous findings. High levels of HbA1c, NTproBNP, hsCRP, and increasing waist circumference were independent predictors of LV systolic reserve.

## 1. Introduction

Globally, the incidence of type 2 diabetes mellitus (T2DM) is increasing. Patients with T2DM have twice the risk of developing cardiovascular disease (CVD) compared with healthy individuals, resulting in an increased risk of death from cardiovascular complications (1). In patients with T2DM, the most common complication and the leading cause of death is diabetic cardiomyopathy (DMCM) (2). In 1972, the concept of DMCM was proposed by Rubler et al., defining that DMCM belonged to a kind of myocardial lesion independent of coronary atherosclerotic heart disease, hypertension, and valvular disease (3). The progression of DMCM eventually results in chronic heart failure (HF). Early notice of subclinical HF in T2DM patients can effectively identify the high-risk patients for cardiovascular complications (4).

Using conventional echocardiography, the first hallmark of DMCM was previously thought to be diastolic dysfunction and was described as typical HF with preserved ejection fraction (EF). However speckle-tracking imaging (STI) could detect subtle changes in LV systolic function earlier than the decrease in the LVEF (5). Consequently, the onset of DMCM is concealed, early LV dysfunction in asymptomatic T2DM patients is subtle, and in a subclinical state, LV function may remain unchanged at rest. Exercise can increase the load of blood flow and increase the work done by LV to maintain the increase of adaptive cardiac output. This can also affect LV reserve function, which is the ability to increase LV function as the body's metabolic requirements increase. Impaired LV reserve function is an early manifestation of various types of HF. Therefore, assessment of LV systolic and diastolic reserve during exercise in combination with STI in patients with asymptomatic T2DM may reveal subclinical myocardial dysfunction and identify early-stage HF.

The identification of biomarkers that accurately predict HF and cardiovascular events in T2DM could improve patient management, aid clinical trials, and highlight novel pathogenesis and therapeutic targets, leading to further improvements in clinical practice (6). Thus, investigating the correlation between LV reserve function and serum biological parameters might provide ideas for the management of T2DM to delay or prevent LV function impairment.

The aims of this study were (1) to explore the clinical application of treadmill ESE combined with STI in assessing LV reserve function, and (2) to analyze the association of serum biological parameters and LV reserve function, which may facilitate early identification of the changes of LV function in asymptomatic T2DM patients.

## 2. Materials and methods

### 2.1. Study population

In a cross-sectional study design, we prospectively enrolled asymptomatic patients with T2DM and healthy controls during the period from September 2021 to July 2022. T2DM was diagnosed according to World Health Organization criteria (7). The exclusion criteria for both populations included LVEF ≤ 50%, any existing CVD, T2DM-related complications (such as kidney disease, neurological disorder and retinopathy), severe obesity and poor echogenicity. None of the subjects had epicardial coronary artery disease as evidenced by coronary angiography and no symptoms (such as difficulty breathing or chest pain during exercise). A total of 100 asymptomatic T2DM patients and 50 healthy control subjects entered the study. All subjects completed clinical examinations that included baseline 12-lead electrocardiography (ECG), and thorough conventional echocardiography at rest and immediately after treadmill exercise (images acquired within 90 s).

Obtaining the basic data for all subjects including gender, age, mass, waist circumference, neck circumference, the waist–hip circumference ratio (WHR), hip circumference, smoking history, drinking history, and resting blood pressure. Serum biological data were collected during fasting and included the measurement of glucose (Glu), glycated hemoglobin (HbA1c), total cholesterol (TC), triglycerides (TG), high- and low-density lipoprotein cholesterol (HDL-C, LDL-C), high-sensitive C-reactive protein (hsCRP), effective glomerular filtration rate (eGFR), homeostasis model assessment of insulin resistance (HOMA-IR), and N-terminal pro-brain natriuretic peptide (NTproBNP). HOMA-IR is the product of fasting serum glucose and fasting serum insulin, calculated as fastingserumglucose(mmol/L)×fastingseruminsulin/22.5 (8).

The study was approved by the local ethics committee and was conducted in accordance with institutional policies, national legal requirements, and the revised principles of the Declaration of Helsinki.

### 2.2. Exercise protocol

Using standard Bruce protocols, each subject completed a symptom-limited treadmill (TMX-425, Full Vision Inc, Kansas, USA) exercise test (9). Subjects were advised to exercise to exhaustion. Blood pressure and a 12-lead electrocardiogram were measured and monitored at rest and again at 2-min intervals throughout the exercise examination, at maximum effort, and after exercise. The exercise test was terminated if the following criteria were reached: symptoms or arrhythmias, hypertension (220/120 mmHg), symptomatic hypotension (> 40 mmHg reduction), and significant ST-segment change (ST-segment depression or elevation >1 mm in two consecutive leads).

### 2.3. Echocardiography recordings and analysis

All subjects underwent standard conventional echocardiography using an ultrasound system Vivid E95 equipped with an M5S 3.5-MHz transducer (GE Vivid E95, Vingmed Ultrasound, Horten, Norway) at rest and immediately after exercise in the left lateral decubitus position. Briefly, at rest and immediately after exercise, at least five periods of two-dimensional images(frame rate >70 frames/second) were obtained from the apical four-, three-, and two-chamber sections and digitally stored.

Both groups complete a comprehensive 2D echocardiographic assessment at rest, according to guidelines of the American Society of Echocardiography(ASE) and the European Association of Cardiovascular Imaging(EACVI), all conventional echocardiographic and Doppler measurements of LV function were analyzed (10). LVEF was calculated using the Simpson biplane method. In the apical four-chamber view, peak early (E wave), atrial (A wave) transmitral flow velocity, and myocardial systolic (s′), early diastolic (e′), and atrial (a′) velocity were obtained using pulsed-wave Doppler (PW) and tissue Doppler velocity imaging (TDI). Simultaneously, we recorded E wave deceleration time (EDT) and LV isovolumic relaxation time (IVRT). All parameters were averaged over 3 consecutive cardiac cycles.

**Figure 1:**
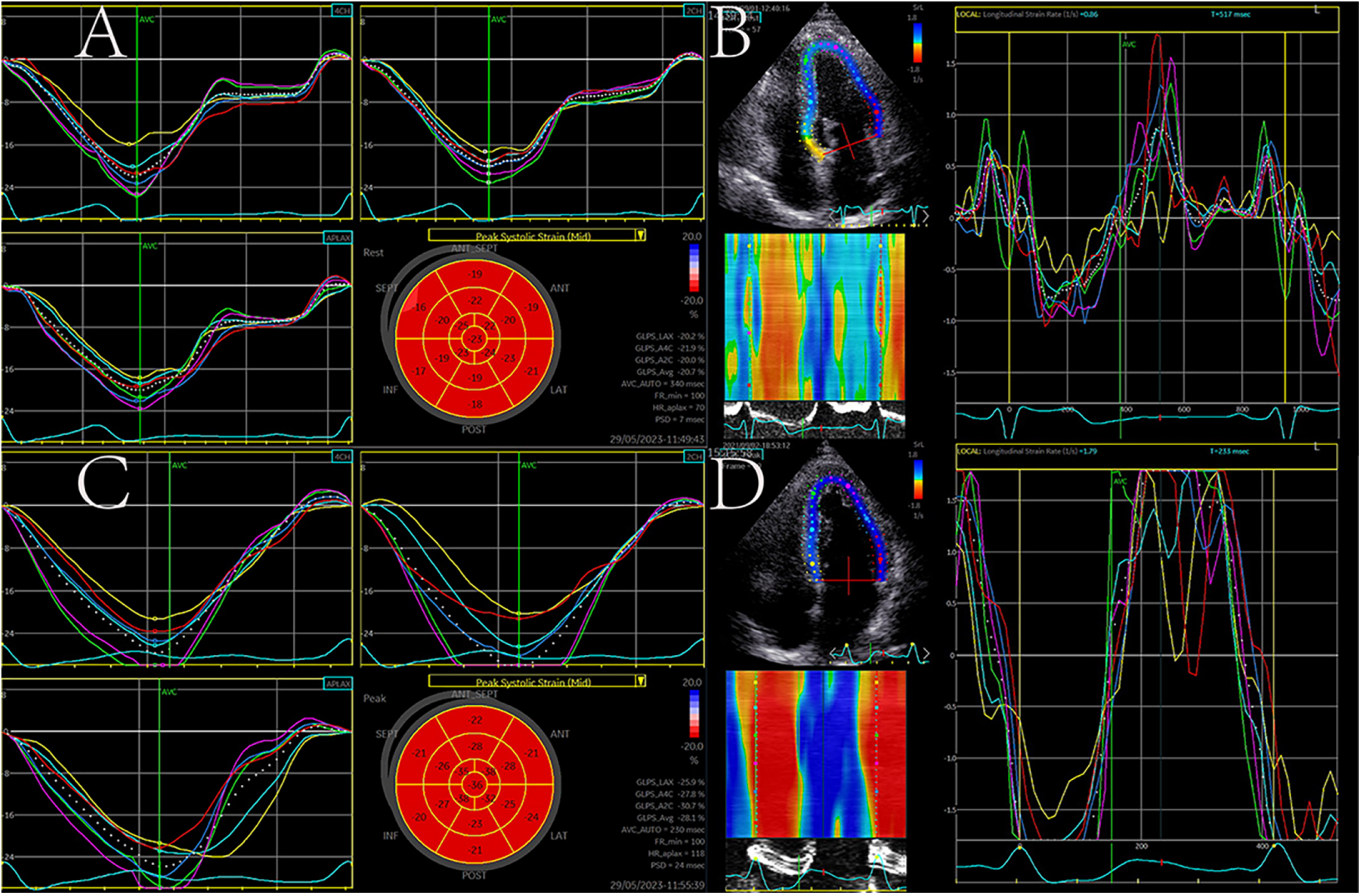
Left ventricular (LV) longitudinal strain (LS) and strain rate (SR) analysis at rest (**A,B**) and post-exercise (**C,D**). GLS and SR both increased after exercise.

Electrocardiogram-triggered echocardiographic data were collected for 2D strain analysis with EchoPac (EchoPAC version 204, GE Healthcare, Horten, Norway). Using Automated Function Imaging to analyze the LV longitudinal function in three apical views (four, three, and two chambers). For optimal tracking results, the speckle region of interest was carefully modified. Segments that did not track adequately were manually readjusted, and if this was ineffective, they were removed from future research. Peak global longitudinal strain (GLS), peak systolic SR (SRs), peak early diastolic SR (SRe), and peak late diastolic SR (SRa) for the LV myocardium were determined (Figure 1). The mean LV GLS/SR was calculated from the three individual apical GLS/SR curves.

### 2.4. Assessment of LV reserve function

The difference between resting and post-exercise LVEF, GLS, SRs, and s′ values were used to compute the LV systolic reserve function (expressed as ΔEF, ΔGLS, ΔSRs, and Δs′, respectively). The difference between resting and post-exercise E, A, EDT, IVRT, e′, a′, E/e′, SRe, and SRa was used to compute the LV diastolic reserve function (expressed as ΔE, ΔA, ΔEDT, ΔIVRT, Δe′, Δa′, ΔE/e′, ΔSRe, and ΔSRa, respectively).

### 2.5. Statistical analysis

Statistical analyses were conducted using SPSS for Windows (IBM SPSS Statistics, version 26), and all continuous variables in the study were presented as mean ± standard deviation or median and interquartile range. Categorical variables are expressed as frequencies and percentages. The normality of all continuous variables was evaluated by the Shapiro-Wilk test to satisfy the assumptions of subsequent tests. For data that conformed to normal distribution, an independent-sample *t*-test was adopted to perform the comparison between two groups, and a paired *t*-test was used for within-group comparisons at rest and after exercise; for data that did not conform to normal distribution, a Mann-Whitney U test was adopted to perform the comparison between two groups, and Wilcoxon rank sum test was adopted for comparisons within a group. The Chi-square test was used to evaluate categorical variables. For correlation analyses, Pearson correlation was used for data that conformed to normal distribution, and Spearman correlation was used for data that did not conform to normal distribution. Independent influence factors of LV reserve function were analyzed by stepwise multivariable regression. *P *< 0.05 was considered to be statistically significant.

### 2.6. Intra-observer and inter-observer variability analysis

To evaluate the reliability of STI-related parameters at rest and immediately after exercise, we randomly selected 10 subjects. The first and second researchers independently re-analyzed the images one week after the initial analysis, without knowledge of the original results. This study was done to assess inter- and intra-observer variability by intra-class correlation(ICC). The intra-observer and interobserver ICC of GLS at rest were 0.94 and 0.93, respectively. The intra-observer and interobserver ICC of GLS after exercise were 0.93 and 0.92, respectively.

## 3. Results

### 3.1. Baseline clinical characteristics

**Figure 2:**
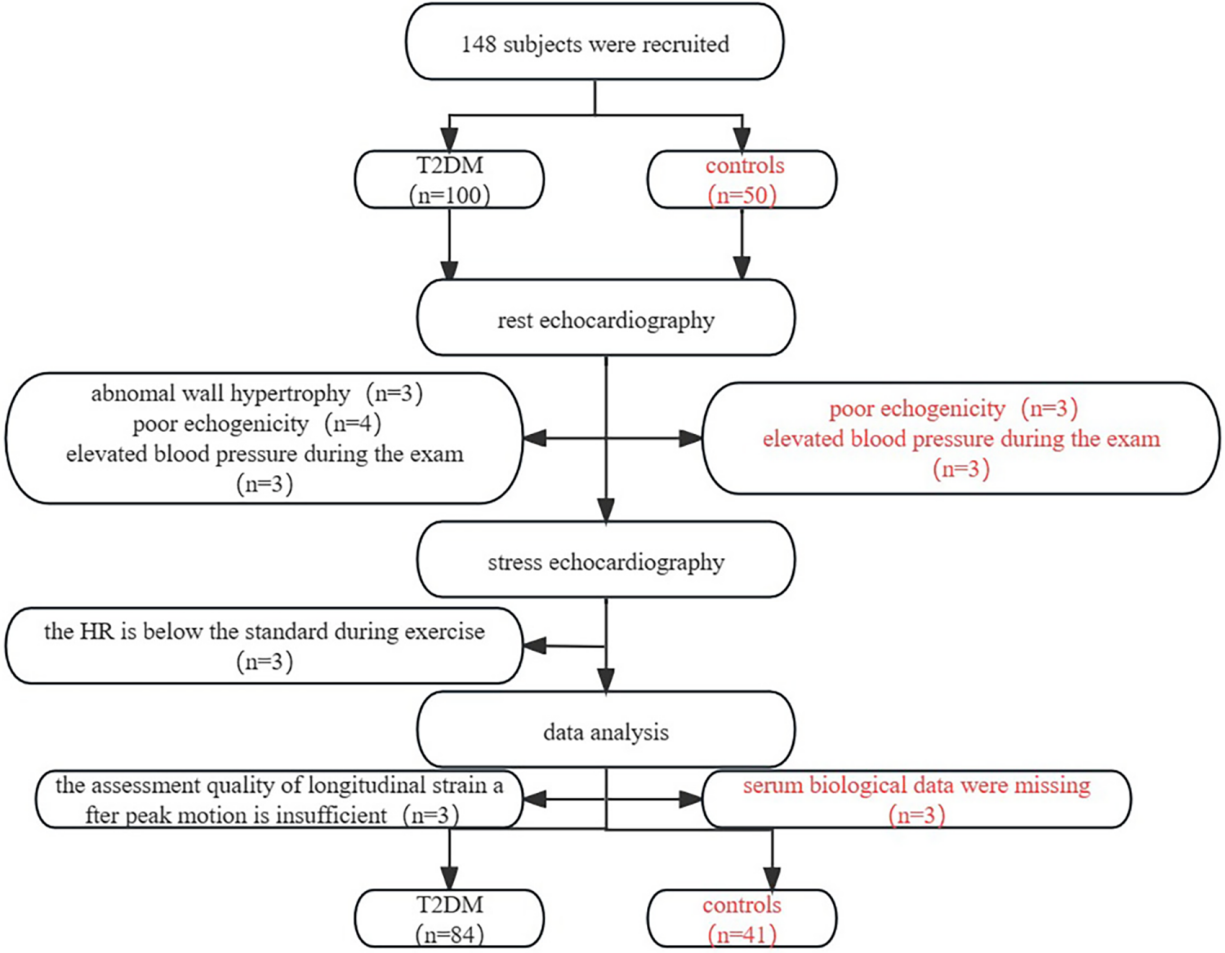
Flow diagram of participant selection.

This study involved 100 asymptomatic T2DM patients and 50 control subjects, after which 84 asymptomatic T2DM patients and 41 control subjects entered the analysis. The selection criteria are shown in the flowchart Figure 2. During the exercise, six asymptomatic T2DM patients experienced mild ST-segment depression. None of the subjects discontinued the testing due to contraindications.84 patients with T2DM (mean age: 58.02 ± 8.54 years; 54% male) and 41 healthy controls (mean age: 56.88 ± 8.95 years; 49% male). Age, gender, smoking and drinking in T2DM group were similar to the controls (*P *> 0.05). The T2DM group had larger neck, waist, and hip circumferences, as well as a higher WHR than the healthy controls (*P *< 0.05). For biological parameters, TC, TG, LDL-C, HDL-C, hsCRP, and eGFR in T2DM group were not different from the control group (*P *> 0.05). However, the T2DM group had higher levels of Glu, HbA1c, HOMA-IR, and NTproBNP compared with those in the control group (*P *< 0.05). Table 1 presents the specific clinical data, outcome measures, and medication usage for both groups.

**Table 1:** Clinical characteristics and 2D echocardiographic characteristics.

	Control subjects	Patients with T2DM	*P*-value
Demographic characteristics			
Gender M/F	20/21	45/39	0.615
Age (years)	56.88 ± 8.95	58.02 ± 8.54	0.471
Neck circumference (cm)	33.70 ± 3.16	35.19 ± 2.87	**0** **.** **011** *
Waist circumference (cm)	82.05 ± 9.65	87.59 ± 8.45	**0** **.** **002** **
WHR	0.873 ± 0.062	0.908 ± 0.057	**0** **.** **003** **
Hip circumference (cm)	93.75 ± 5.88	96.46 ± 6.67	**0** **.** **031** *
SBP (mmHg)	120.29 ± 13.26	130.50 ± 15.28	**<0** **.** **001** ***
DBP (mmHg)	71.83 ± 10.52	74.26 ± 9.91	0.209
Drinking (%)	75.6%	78.6%	0.709
Smoking (%)	78.0%	81.0%	0.703
Biological parameters			
NTproBNP (pg/ml)	28.00 (23.00, 130.25)	116.00 (89.00, 178.00)	**<0** **.** **001** ***
HbA1c (%)	5.38 ± 0.33	6.95 ± 1.38	**<0** **.** **001** ***
HOMA-IR	1.23 (0.84, 1.58)	2.24 (1.30, 3.11)	**<0** **.** **001** ***
Glu (g/L)	5.46 ± 0.33	8.22 ± 2.74	**<0** **.** **001** ***
Insulin (mUI/L)	5.00 (3.33, 6.43)	5.30 (3.80, 9.40)	0.108
hsCRP	0.00 (0.00, 0.86)	0.00 (0.00, 0.90)	0.290
TC (g/L)	4.98 ± 0.82	4.75 ± 1.01	0.225
TG (g/L)	1.11 (0.89, 1.73)	1.28 (0.91, 1.65)	0.554
LDL-C (g/L)	2.78 ± 0.67	2.60 ± 0.73	0.195
HDL-C (g/L)	1.46 ± 0.28	1.46 ± 0.40	0.903
eGFR	99.11 ± 12.36	99.25 ± 11.93	0.951
2D echocardiographic parameters			
EDV (ml)	66.05 ± 16.71	68.05 ± 16.71	0.283
ESV (ml)	24.44 ± 5.31	25.31 ± 7.09	0.491
LV Mass (g)	91.43 ± 11.50	95.75 ± 12.34	0.067
LVEF %	0.63 ± 0.04	0.63 ± 0.04	0.707
IVSd (mm)	7.00 (6.50, 8.00)	9.00 (8.00, 10.00)	**<0** **.** **001** ***
LVPWd (mm)	7.00 (7.00, 8.00)	8.00 (7.00, 9.00)	**0** **.** **010** *
Treadmill ESE time(min)	8:04 ± 1:98	8:10 ± 1:70	0.864
Symptoms during treadmill ESE			
Mild ST-segment depress	2 (4.8%)	6 (7.1%)	**<0** **.** **001** ***
Premature atrial contraction	3 (7.3%)	8 (9.5%)	**<0** **.** **001** ***
Premature ventricular contraction	1 (2.4%)	5 (5.9%)	**<0** **.** **001** ***
Symptoms of dyspnoea	0	0	/
RWMA	0	0	/
Medication			
Antidiabetic drugs	0	46 (54.7%)	/
Antidyslipidemic drugs	3 (7.3%)	22 (26.1%)	**<0** **.** **001** ***
Antihypertensive drugs	0	12 (14.3%)	/

M/F, males/females; WHR, the waist–hip ratio; SBP, systolic blood pressure; DBP, diastolic blood pressure; NTproBNP, N-terminal pro-B-type natriuretic peptide; HbA1c, glycated hemoglobin; HOMA-IR, homeostasis model assessment of insulin resistance; Glu, Glucose; hsCRP, high-sensitive C-reactive protein; TC, total cholesterol, TG, triglycerides; HDL-C and LDL-C, high- and low-density lipoproteins cholesterol; eGRF, effective glomerular filtration rate; EDV, end-diastolic volume; ESV, end-systolic volume; LVEF, left ventricular ejection fraction; IVSd, left ventricular end-diastolic interventricular septal thickness; LVPWd, left ventricular posterior wall thickness end-diastole; Treadmill ESE time, treadmill exercise stress echocardiography time; RWMA, regional wall motion abnormality.

* *P* < 0.05.

** *P* < 0.01.

*** *P *< 0.001.

Statistically significant *P* values are shown in bold.

### 3.2. Echocardiographic characteristics

Table 1 presents the parameters of conventional 2D echocardiographic in the two groups. The difference in LV end-diastolic volume (LVEDV) and LV end-systolic volume (LVESV) between the two groups were not statistically significant (*P *> 0.05). LVEF in the T2DM group was comparable to that in the control group (*P *> 0.05).

### 3.3. LV function at rest and after exercise

**Figure 3:**
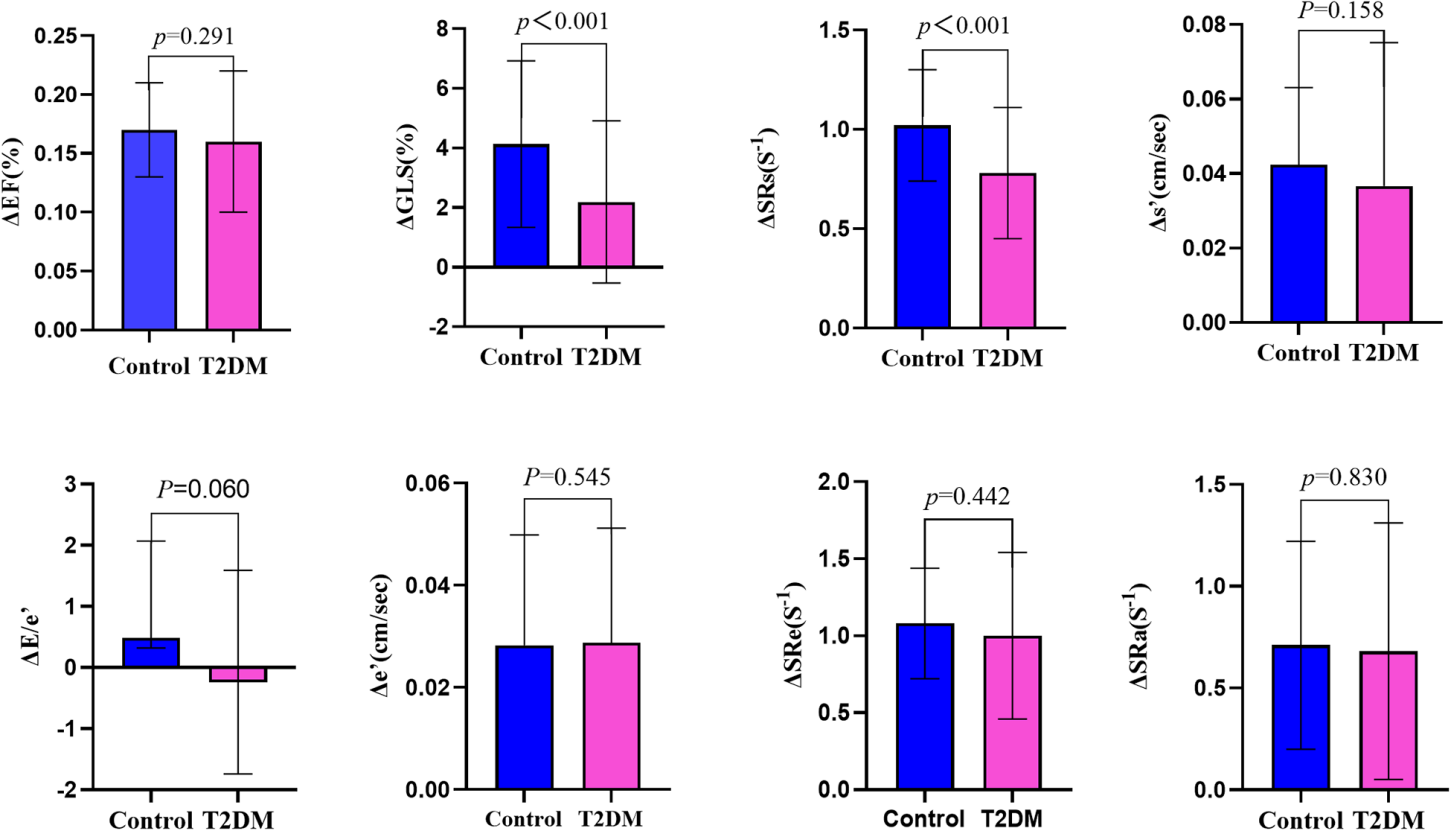
Systolic reserve and diastolic reserve parameters were compared between patients with asymptomatic type 2 diabetes mellitus and healthy controls. Δ, delta value; EF, ejection fraction; GLS, global longitudinal strain; SRs, peak systolic longitudinal strain rate; s′, systolic mitral annulus tissue velocity; e′, early diastolic mitral annulus tissue velocity; SRe, early diastolic longitudinal strain rate; SRa, late diastolic longitudinal strain rate.

Table 2 summarizes the resting and post-exercise parameters of LV function for both groups. The difference in E and s′ between the two groups were not significant (*P *> 0.05) at rest, but the T2DM group had significantly higher levels of A, a′, and E/e′ (*P *< 0.05) at rest. Compared with the control group, e′ were markedly decreased in the patients with T2DM (*P < *0.05) at rest, but these parameters were still within the normal range according to current guideline (10). During exercise, these parameters of diastolic function increased in both groups. After exercise, the differences in E, A, and E/e′ between the two groups were not significant (*P *> 0.05). ΔE, ΔA, ΔEDT, ΔIVRT, Δe′, Δa′, ΔE/e′, ΔSRe, and ΔSRa did not significantly differ between the two group (Table 3 and Figure 3), indicating that the diastolic reserve in the T2DM group was preserved as in the control group.

**Table 2:** Hemodynamic and echocardiographic data during ESE.

	Rest			Post-exercise		
	Control subjects	Patients with T2DM	*P*-value	Control subjects	Patients with T2DM	*P*-value
Longitudinal function						
GLS (%)	19.27 ± 2.42	18.83 ± 2.72	0.384	23.55 ± 3.23	21.02 ± 2.95	0.001***
SRs (s^−1^)	1.05 ± 0.16	1.06 ± 0.15	0.767	2.08 ± 0.26	1.84 ± 0.36	0.001***
SRe (s^−1^)	1.37 ± 0.41	1.16 ± 0.36	0.005**	2.46 ± 0.52	2.17 ± 0.55	0.005**
SRa (s^−1^)	1.03 ± 0.20	1.18 ± 0.26	0.002**	1.74 ± 0.57	1.87 ± 0.68	0.308
Pulsed-wave						
E (cm/sec)	0.74 ± 0.14	0.70 ± 0.17	0.230	1.05 ± 0.29	0.96 ± 0.24	0.068
A (cm/sec)	0.70 ± 0.18	0.83 ± 0.18	<0.001***	1.00 ± 0.25	1.08 ± 0.26	0.103
EDT(msec)	212.14 ± 6.96	211.61 ± 5.30	0.954	169.75 ± 7.76	158.13 ± 5.14	0.206
IVRT (msec)	97.02 ± 1.97	95.97 ± 2.12	0.743	82.85 ± 1.79	78.92 ± 2.23	0.197
TDI parameters						
e′(cm/sec)	0.10 ± 0.02	0.08 ± 0.02	<0.001***	0.13 ± 0.03	0.11 ± 0.03	0.002**
a′ (cm/sec)	0.09 ± 0.02	0.10 ± 0.02	<0.001***	0.13 ± 0.03	0.15 ± 0.03	<0.001***
s′ (cm/sec)	0.09 ± 0.02	0.09 ± 0.03	0.710	0.13 ± 0.03	0.13 ± 0.03	0.500
E/ e′	7.82 ± 2.15	9.20 ± 2.78	0.004*	8.56 ± 2.40	9.18 ± 3.03	0.261
Hemodynamics						
HR	84.71 ± 13.74	85.00 ± 11.99	0.903	156.95 ± 19.08	153.95 ± 16.91	0.374
SBP (mmHg)	120.29 ± 13.26	130.50 ± 15.28	0.001***	167.73 ± 29.88	189.61 ± 25.39	0.001***
DBP (mmHg)	71.83 ± 10.52	74.26 ± 9.91	0.209	79.44 ± 13.79	80.60 ± 16.46	0.699

GLS, global longitudinal strain; SRs, peak systolic longitudinal strain rate; SRe, early diastolic longitudinal strain rate; SRa, late diastolic longitudinal strain rate; E, mitral flux early diastolic wave; A, mitral flux late diastolic wave; EDT, E wave deceleration time; IVRT, isovolumic relaxation time; e′, early diastolic mitral annulus tissue velocity; s′, systolic mitral annulus tissue velocity; HR, heart rate; SBP/DBP, systolic/diastolic blood pressure.

* *P* < 0.05.

** *P* < 0.01.

*** *P* < 0.001.

Statistically significant *P* values are shown in bold.

**Table 3:** Lv reserve function assessment.

	Control subjects	Patients with T2DM	*P*-value
LV systolic reserve			
ΔEF (%)	0.17 ± 0.04	0.16 ± 0.06	0.291
Δs′ (cm/sec)	0.042 ± 0.03	0.038 (0.028, 0.050)	0.158
ΔGLS (%)	4.13 ± 2.79	2.19 ± 2.72	**<0.001**
ΔSRs (%)	1.02 ± 0.28	0.78 ± 0.33	**<0.001**
LV diastolic reserve			
ΔE (cm/sec)	0.26 (0.19, 0.42)	0.25 ± 0.02	0.518
ΔA (cm/sec)	0.30 (0.19, 0.37)	0.25 ± 0.02	0.286
ΔEDT(msec)	−42.38 ± 9.90	−53.48 ± 5.71	0.302
ΔIVRT(msec)	−16.70 ± 2.45	−14.17 ± 1.90	0.433
Δe′ (cm/sec)	0.025 (0.015, 0.040)	0.029 ± 0.002	0.544
Δa′ (cm/sec)	0.038 ± 0.004	0.038 ± 0.003	0.829
ΔE/e′	0.49 (−0.17, 1.58)	−0.25 (−1.49, 1.84)	0.060
ΔSRa (s^−1^)	0.71 ± 0.51	0.68 ± 0.63	0.830
ΔSRe (s^−1^)	1.08 ± 0.36	1.00 ± 0.54	0.442

Δ, delta value; EF, ejection fraction; s′, systolic mitral annulus tissue velocity; GLS, global longitudinal strain; SRs, peak systolic longitudinal strain rate; E, mitral flux early diastolic wave; A, mitral flux late diastolic wave; EDT, E wave deceleration time; IVRT, isovolumic relaxation time; e′, early diastolic mitral annulus tissue velocity; a′, lately diastolic mitral annulus tissue velocity, SRa, late diastolic longitudinal strain rate; SRe, early diastolic longitudinal strain rate.

Statistically significant *P* values are shown in bold.

At rest, LVGLS and SRs in asymptomaticT2DM patients were similar to those in healthy individuals (*P *> 0.05), but significant differences were revealed after exercise (*P *< 0.05). Although the differences in ΔEF and Δs′ were not significant between the two groups(*P *< 0.05), ΔGLS and ΔSRs were both lower in the T2DM group compared with those in the control group (Table 3 and Figure 3). GLS and SRs were increased in T2DM group during exercise, but compared with the control group, the increase was noticeably less than the control group, indicating a considerably decreased systolic reserve.

**Figure 4:**
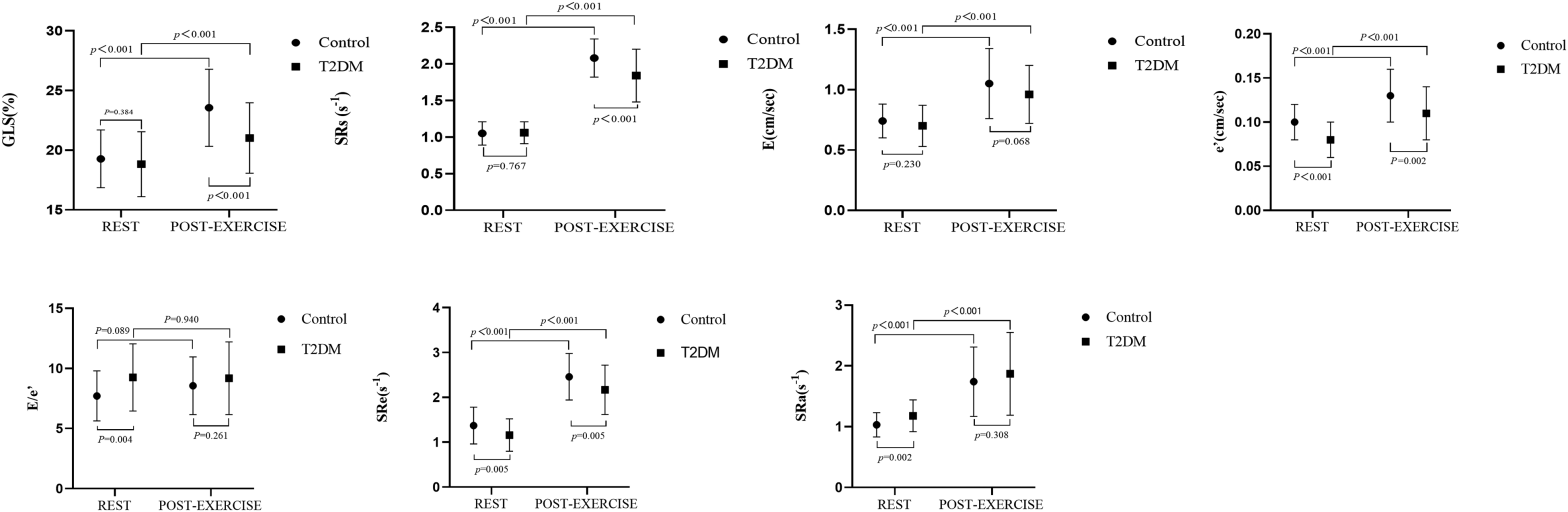
Systolic and diastolic reserve function parameters were compared between and within the two groups at rest and after exercise. In addition to the E/ e′ ratio, systolic and diastolic parameters increased significantly after exercise in both the control and asymptomatic T2DM groups.

Table 4 and Figure 4 illustrate the alterations in parameters associated with systolic and diastolic function at rest and post-exercise, comparing the asymptomatic T2DM group with the control group, as well as within each group. In the control group, the parameters related to diastolic function (E, A, e′, a′, SRa, SRe) exhibited a significant increase, while EDT and IVRT were notably shortened after exercise compared to the resting state. However, there was no significant difference in E/e′ between rest and post-exercise. Additionally, the systolic function parameters (GLS, SRs, and s′) also showed a significant increase after exercise. These findings were similarly observed in the asymptomatic T2DM group. It is suggested that both diastolic and systolic functions responded adequately to exercise in both the control and the asymptomatic T2DM groups.

**Table 4:** Intra-group comparison of echocardiographic data during ESE.

	Control subjects			Patients with T2DM		
	Rest	Post-exercise	*P*-value	Rest	Post-exercise	*P*-value
Longitudinal function						
GLS (%)	19.27 ± 2.42	23.55 ± 3.23	<0.001***	18.83 ± 2.72	21.02 ± 2.95	<0.001***
SRs (s^−1^)	1.05 ± 0.16	2.08 ± 0.26	<0.001***	1.06 ± 0.15	1.84 ± 0.36	<0.001***
SRe (s^−1^)	1.37 ± 0.41	2.46 ± 0.52	<0.001***	1.16 ± 0.36	2.17 ± 0.55	<0.001***
SRa (s^−1^)	1.03 ± 0.20	1.74 ± 0.57	<0.001***	1.18 ± 0.26	1.87 ± 0.68	<0.001***
Pulsed-wave						
E (cm/sec)	0.74 ± 0.14	1.05 ± 0.29	<0.001***	0.70 ± 0.17	0.96 ± 0.24	<0.001***
A (cm/sec)	0.70 ± 0.18	1.00 ± 0.25	<0.001***	0.83 ± 0.18	1.08 ± 0.26	<0.001***
EDT(msec)	212.14 ± 6.96	169.75 ± 7.76	<0.001***	211.61 ± 5.30	158.13 ± 5.14	<0.001***
IVRT(msec)	97.02 ± 1.97	82.85 ± 1.79	<0.001***	95.97 ± 2.12	78.92 ± 2.23	<0.001***
TDI parameters						
e′ (cm/sec)	0.10 ± 0.02	0.13 ± 0.03	<0.001***	0.08 ± 0.02	0.11 ± 0.03	<0.001***
a′ (cm/sec)	0.09 ± 0.02	0.13 ± 0.03	<0.001***	0.10 ± 0.02	0.15 ± 0.03	<0.001***
s′ (cm/sec)	0.09 ± 0.02	0.13 ± 0.03	<0.001***	0.09 ± 0.03	0.13 ± 0.03	<0.001***
E/ e′	7.87 ± 2.15	8.56 ± 2.40	0.089	9.20 ± 2.78	9.18 ± 3.03	0.940

GLS, global longitudinal strain; SRs, peak systolic longitudinal strain rate; SRe, early diastolic longitudinal strain rate; SRa, late diastolic longitudinal strain rate; E, mitral flux early diastolic wave; A, mitral flux late diastolic wave; EDT, E wave deceleration time; IVRT, isovolumic relaxation time; e′, early diastolic mitral annulus tissue velocity; s′, systolic mitral annulus tissue velocity.

Statistically significant *P* values are shown in bold.

### 3.4. Correlates of LV systolic reserve function

**Figure 5:**
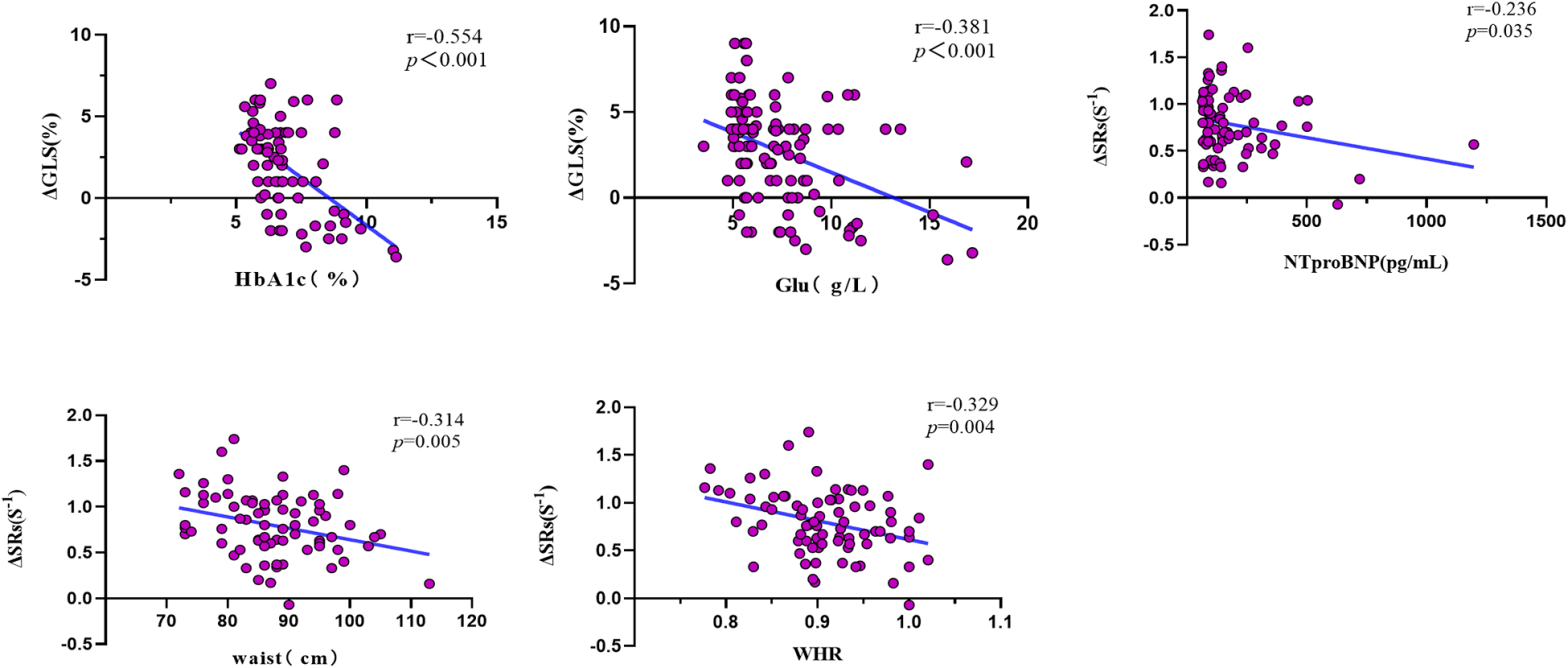
Univariate correlation analysis of LV systolic reserve function with clinical and biological parameters. Δ, delta value; GLS, global longitudinal strain; SRs, peak systolic longitudinal strain rate; HbA1c, glycated hemoglobin; Glu, Glucose; NTproBNP, N-terminal pro-B-type natriuretic peptide; WHR, the waist–hip ratio.

In Table 5 and Figure 5, the outcomes of the univariate and multivariate regression analysis are shown. ΔGLS was good correlated with HbA1c and glucose, and ΔSRs was mildly correlated with waist circumference and WHR, and ΔSRs was weak correlated with NTproBNP. ΔGLS was significantly independently correlated with HbA1c (*β *= −0.614, *P *< 0.001), and ΔSRs was modest independently correlated with NTproBNP (*β* = −0.262, *P *= 0.027), waist circumference (*β* = −0.299, *P *= 0.013), and hsCRP (*β* = −0.245, *P* = 0.039) through the stepwise multiple linear regression analysis.

**Table 5:** Univariate and multivariate correlation analysis of systolic reserve parameters with clinical and biologic parameters.

	Univariate				Multivariate			
	ΔGLS		ΔSRs		ΔGLS		ΔSRs	
	*r*	*p*	*r*	*p*	*β*	*p*	*β*	*p*
Age	−0.017	0.883	−0.120	0.287	0.004	0.968	−0.100	0.397
LV Mass	−0.165	0.150	0.072	0.532	−0.128	0.214	0.062	0.603
neck	0.030	0.794	−0.127	0.270	−0.008	0.939	−0.074	0.595
waist	0.029	0.803	**−0** **.** **314**	**0** **.** **005** **	0.074	0.475	**−0** **.** **299**	**0** **.** **013** *
hip	0.191	0.096	−0.147	0.202	0.105	0.329	0.042	0.829
HbA1c	**−0** **.** **554**	**0** **.** **000** ***	0.105	0.364	**−0** **.** **614**	**0** **.** **000** ***	0.075	0.537
eGFR	−0.058	0.616	0.127	0.268	0.052	0.616	−0.007	0.961
Glu	**−0** **.** **381**	**0** **.** **001** **	0.171	0.143	0.234	0.208	0.081	0.506
TC	−0.045	0.693	0.132	0.251	−0.072	0.485	0.123	0.297
LDL-C	−0.136	0.234	0.157	0.171	−0.078	0.460	0.177	0.131
HDL-C	0.199	0.080	−0.028	0.808	−0.028	0.800	−0.040	0.743
NTproBNP	0.101	0.372	**−0** **.** **236**	**0** **.** **035** *	0.001	0.993	**−0** **.** **262**	**0** **.** **027** *
hsCRP	−0.157	0.173	−0.199	0.083	−0.085	0.417	**−0** **.** **245**	**0** **.** **039** *
Insulin	0.160	0.175	0.013	0.912	0.009	0.933	−0.048	0.691
TG	0.041	0.724	0.056	0.623	0.059	0.569	0.050	0.681
WHR	−0.182	0.112	**−0** **.** **329**	**0** **.** **004** **	0.009	0.931	−0.033	0.849
HOMA-IR	0.020	0.869	0.006	0.959	0.019	0.858	−0.094	0.438

Δ, delta value; HbA1c, glycated hemoglobin; eGFR, glomerular filtration rate. Glu, Glucose; TC, total cholesterol; HDL and LDL, high- and low-density lipoproteins; NTproBNP, N-terminal pro-B-type natriuretic peptide; hsCRP, high-sensitive C-reactive protein; TG, triglycerides; WHR, the waist–hip ratio; HOMA-IR, homeostasis model assessment of insulin resistance.

* *p* < 0.05.

** *p* < 0.01.

*** *p* < 0.001.

Statistically significant *P* values are shown in bold.

## 4. Discussion

This study provides a thorough analysis of LV reserve function in asymptomatic T2DM patients, which is crucial for understanding the development of HF. Furthermore, we looked into the link between biomarkers and myocardial function in T2DM, enabling early detection of changes in LV function. A first principal outcome of the study was that asymptomatic T2DM patients have the similar GLS and SRs at rest to the controls. In contrast, compared to the control group GLS and SRs rose less during exercise in the T2DM group, whereas the changes in E/e′, E, A, EDT, IVRT, a′, e′, SRe, and SRa during exercise were similar in two groups. This suggests that LV systolic reserve impairment might appear early before diastolic reserve impairment. Our second major finding was that the primary contributors to these patients' decreased systolic response to treadmill ESE are high levels of HbA1c, NTproBNP, hsCRP and increased waist circumference, especially increased HbA1c level.

T2DM is recognized as a separate CVD risk factor (11). T2DM and HF often coexist, and the presence of one enhances the incidence and severity of the other (12, 13). Left HF is particularly significant for patient prognosis, as the LV reserve function can indicate the progression from left HF to refractory or end-stage HF. Research has shown that the initial pathological change in HF patients is a decrease in cardiac reserve (14–16). Early detection of abnormal LV reserve function in patients with T2DM is beneficial for the early identification of high-risk individuals with HF, and combined with relevant biological markers, will facilitate active interventions to prevent HF.

### 4.1. Analysis of LV diastolic function and diastolic reserve in T2DM

The complex factors of DMCM can lead to structural changes in the myocardium. LV hypertrophy and myocardial fibrosis are the earliest morphological changes that occur in patients with early and asymptomatic diabetes and can lead to diastolic dysfunction. Abnormal ventricular reserve function indicates subclinical ventricular dysfunction early. The ability of the LV to strengthen its diastolic function to preserve normal filling pressure during exercise is referred to as LV diastolic reserve function. Several studies of diastolic reserve in T2DM patients, which showed the differences in e′ and E/e′ were not significant between the two groups at rest. e′ velocity rose significantly less and the E/e′ ratio increased significantly more in the T2DM group during exercise, suggesting impaired diastolic reserve in this population (17, 18). Hence, in the early phases of diastolic dysfunction, such as that found in DMCM, a reduced diastolic reserve may be observed (19). However, our study showed contrasting results. Although some diastolic function parameters in the asymptomatic T2DM group have been significantly different from those in the control group at rest, it is not yet possible to diagnose diastolic dysfunction according to current guidelines (10). Regardless of whether it was the control group or the asymptomatic T2DM group, both E and e′ increased after exercise. The increase in these two parameters was similar, indicating minimal changes in E/e′ before and after exercise in both groups. Additionally, our study revealed that E, e′, and E/e′ increases during exercise were comparable in asymptomatic T2DM patients to controls, and during exercise the SRe and SRa rises were comparable between the two groups. During exercise, increased sympathetic tone and an aggravated LV suction action result in a higher relaxation rate. This improves the E wave and sustains LV filling volumes despite a shorter diastolic filling time, with no appreciable pressure increase in the left atrium (20). Both E and e′ velocities increased correspondingly in patients with normal myocardial relaxation, resulting in little change in the E/e′ ratio. However, e′ velocity changed less than E velocity in patients with abnormal myocardial relaxation, causing an increased E/e′ ratio with exercise (21). In contrast to previous studies, our study found that patients with asymptomatic T2DM demonstrated normal myocardial relaxation and did not exhibit any noticeable impairment of diastolic reserve.

### 4.2. Analysis of LV systolic function and systolic reserve in T2DM

LV systolic function is instrumental in CVD. In our study, changes in LVEF were similar between the two groups both at rest and after exercise. Patients with asymptomatic T2DM showed similar results as the control group for LVGLS and SRs at rest. However, after exercise, LVGLS and SRs were significantly impaired in the T2DM group. Previous studies revealed that LVEF was not a sensitive index to identify subclinical LV systolic dysfunction, and a sensitive and feasible approach is to assess LV systolic function by GLS using STI (22). It is difficult to identify modest impairment of LV systolic function by resting deformation imaging (23), and stress echocardiography (SE) could be helpful in revealing subclinical abnormalities. SE is frequently employed to evaluate LV systolic reserve in patients with coronary artery disease and patients with conditions such valvular diseases (24, 25), hypertrophic cardiomyopathy (26), and hypertensive heart disease (27). Systolic reserve refers to the ventricle's capacity to respond to stress and serves as a disease outcome predictor (28). Decreased systolic reserve is considered an early sign of LV dysfunction (29). Previous studies have not specifically examined systolic reserve using STI in asymptomatic T2DM. We found significant lower increase in GLS and SRs during exercise in asymptomatic T2DM group than in the controls, indicating that their longitudinal contractile reserve is abnormal, GLS and SRs primarily represent the function of subendocardial myocardial fibers, which are more vulnerable to ischemia and increased wall stress, and whose function is impaired by interstitial fibrosis and abnormal microcirculation (30). Patients with T2DM may experience microvascular dysfunction, which is a common complication of T2DM and can occur in the diabetic heart before macrovascular disease. During exercise, the body requires more oxygen, and microvascular dysfunction can lead to myocardial hypoxia (31). This can affect myocardial behavior during a stress state and may explain the abnormal reserve of systolic function observed in our patients. After loading conditions, the lower deformation measurements in T2DM patients after exercise strongly suggest that there are changes in cardiac intrinsic contraction.

### 4.3. The association of LV systolic reserve function and serum biological parameters

For the prediction of incident HF and cardiovascular events in patients with T2DM and in those with existing HF and T2DM, we looked at how clinical features and serum biological markers affected LV systolic reserve. Based on univariate and multivariate correlation analyses, we identified the independent factors associated with LV systolic reserve. Parameters related to LV systolic reserve function are associated with the markers of diabetes metabolism, visceral adiposity, and the cardiovascular system. In particular, HbA1c, an indicator of glycemic control, showed an independent and significant correlation with ΔGLS.

In diabetic patients, HbA1c offers an indication of glycemic control over a period of two to three months, the standard for good glycemic control is HbA1c < 7% (32). Hyperglycemia is considered as an initiating factor, poor glycemic control can cause a range of negative effects on myocardial metabolism, including disruptions to the excitation–contraction relationship, increased oxidative stress and metabolic substrates. These metabolic disturbances can also activate inflammatory pathways in diabetes. Ultimately, disturbances in glucose and lipid metabolism can lead to cardiac hypertrophy, fibrosis, increased stiffness, and loss of cardiomyocytes (33). Such changes result in significant impairment of LV reserve function. Numerous studies have verified the link between HbA1c and incident cardiac events (34). Each 20 mg/dl rise in blood glucose increases the risk of HF by 25%, and each 1% increase in HbA1c increases the risk by 8% (35,36). According to recent studies found that the greatest decrease of HbA1c levels were beneficial to the improvement of diastolic and systolic functions in patients (37–43). Therefore, in diabetic patients, maintaining appropriate blood glucose control may help lower their risk of myocardial damage and HF.

One of the measures of abdominal adiposity is waist circumference which was found to be independently and mildly associated with LV systolic reserve function in our study. Previous studies have found that increased abdominal obesity is independently associated with impaired left ventricular systolic function, regardless of the presence of systemic obesity (44). The mechanism may be that abdominal obesity negatively affects myocardial function due to its association with inflammatory cytokines, insulin resistance and hyperinsulinemia, as well as lipotoxicity resulting from lipid accumulation in cardiac tissue (45). Several studies have highlighted that weight loss plays an important role in the reversibility of impaired systolic and diastolic function in diabetic patients (37, 46–48). NTproBNP can be utilized to assess diabetic patients for subclinical LV impairment (49–51). NTproBNP is secreted by ventricular myocytes due to increased wall tension. Therefore, an important factor determinant of NTproBNP is wall stress, which is determined by LV diastolic filling pressure, wall thickness and LV diastolic diameter. This may explain the independent but weak association of NTproBNP with LV systolic reserve function in our study. In addition, our study found that hsCRP, an important biomarker of inflammation, was only independently associated with LV systolic reserve. Evidence from previous studies has suggested that hsCRP is associated with metabolic syndrome and DM, and can be used as a clinical tool to assess cardiovascular risk, showing strong predictive power in patients known to have CVD (52). However, current studies using indicators of inflammatory that have exclusively considered atherosclerotic cardiovascular events without taking heart failure into account have failed to successfully predict risk (6).

The collective findings of these studies emphasize the significance of effectively managing HbA1c, waist circumference, NTproBNP and hsCRP in the treatment of DMCM and in preventing the progression of DMCM from subclinical to overt stages. Particularly, maintaining good glycemic control is crucial.

### 4.4. Research limitations

The limitations of this study are as follows:

1. This study was a single-center study with a small sample size resulting in a high standard deviation of the measurement parameters. In addition, it is important to note that the results of this study are based on hypotheses.
2. LV function is the result of multi-directional deformation (longitudinal, circumferential, and radial direction) interacting with torsional mechanics. This study solely looked at longitudinal strain, and circumferential/radial strain and LV torsion angle (twist) need to be taken into consideration in future work. Furthermore, since the heart has a three-dimensional (3D) structure, its motion is also 3D. However, some of this information is inevitably lost because this research is based on 2D STI, resulting in a less comprehensive and less accurate assessment of myocardial strain.

## 5. Conclusion

In this study, we found that asymptomatic patients with T2DM exhibited impaired systolic reserve function, while diastolic reserve function remained preserved. This finding contradicts previous suggestions that diastolic function is impaired first in DMCM. These results suggest that combining treadmill ESE with STI might be valuable in detecting subtle myocardial injury in asymptomatic T2DM patients. Furthermore, HbA1c, waist circumference, NTproBNP and hsCRP are independent predictors of the blunted systolic function which results in an inadequate response to treadmill ESE in these patients. Good control of these parameters, particularly HbA1c levels, might be beneficial for LV systolic reserve function.

## Data Availability

The original contributions presented in the study are included in the article/Supplementary Material, further inquiries can be directed to the corresponding authors.
